# Radiomics of Patients with Locally Advanced Rectal Cancer: Effect of Preprocessing on Features Estimation from Computed Tomography Imaging

**DOI:** 10.1155/2022/2003286

**Published:** 2022-03-20

**Authors:** Stefania Linsalata, Rita Borgheresi, Daniela Marfisi, Patrizio Barca, Aldo Sainato, Fabiola Paiar, Emanuele Neri, Antonio Claudio Traino, Marco Giannelli

**Affiliations:** ^1^Unit of Medical Physics, Pisa University Hospital “Azienda Ospedaliero-Universitaria Pisana”, Pisa, Italy; ^2^Radiation Oncology Unit, Pisa University Hospital “Azienda Ospedaliero-Universitaria Pisana”, Pisa, Italy; ^3^Diagnostic and Interventional Radiology, Department of Translational Research, University of Pisa, Pisa, Italy

## Abstract

The purpose of this study was to investigate the effect of image preprocessing on radiomic features estimation from computed tomography (CT) imaging of locally advanced rectal cancer (LARC). CT images of 20 patients with LARC were used to estimate 105 radiomic features of 7 classes (shape, first-order, GLCM, GLDM, GLRLM, GLSZM, and NGTDM). Radiomic features were estimated for 6 different isotropic resampling voxel sizes, using 10 interpolation algorithms (at fixed bin width) and 6 different bin widths (at fixed interpolation algorithm). The intraclass correlation coefficient (ICC) and the coefficient of variation (CV) were calculated to assess the variability in radiomic features estimation due to preprocessing. A repeated measures correlation analysis was performed to assess any linear correlation between radiomic feature estimate and resampling voxel size or bin width. Reproducibility of radiomic feature estimate, when assessed through ICC analysis, was nominally excellent (ICC > 0.9) for shape features, good (0.75 < ICC ≤ 0.9) or moderate (0.5 < ICC ≤ 0.75) for first-order features, and moderate or poor (0 ≤ ICC ≤ 0.5) for textural features. A number of radiomic features characterized by good or excellent reproducibility in terms of ICC showed however median CV values greater than 15%. For most textural features, a significant (*p* < 0.05) correlation between their estimate and resampling voxel size or bin width was found. In CT imaging of patients with LARC, the estimate of textural features, as well as of first-order features to a lesser extent, is appreciably biased by preprocessing. Accordingly, this should be taken into account when planning clinical or research studies, as well as when comparing results from different studies and performing multicenter studies.

## 1. Introduction

Radiomics concerns the management of standard-of-care digital medical images from different modalities (e.g., computed tomography (CT), magnetic resonance (MR), and nuclear medicine (NM)), with the aim of mining from them pathophysiologic changes underlying disease [1]. Specifically, quantitative morphological and textural characteristics of tissue can be obtained from medical images by measuring different mathematical indices, namely, “features.” In addition to other available data from demographics, pathology, blood biomarkers, and genomics, radiomic features can be used for diagnostic, prognostic, or predictive purposes exploiting statistical or machine learning methods [2]. Despite its potential, radiomics is not yet a widely used and well-consolidated tool in clinical practice, since it involves complex processes (e.g., image acquisition and reconstruction, image segmentation and rendering, features estimation, databases and data sharing, classification, and analysis), which need proper application and optimization in order to obtain reliable results [3, 4]. Moreover, accurate classification methods using radiomic features and artificial intelligence (AI) require large data sets. Given that each step of the radiomic workflow can introduce noise (i.e., variability) in radiomic features estimation, robust radiomic features should be obtained for adequately training AI predictive models, mostly when only limited data are available as in many practical applications [5].

Colorectal cancer is the most common gastrointestinal malignancy and the third leading cause of cancer-related death in Western countries. More than half of rectal cancer patients are diagnosed with locally advanced tumors (locally advanced rectal cancer (LARC): T3/T4 tumor and/or positive limphonodes) [6]. For this group of patients, preoperative radiochemotherapy (RTCT) followed by total mesorectal excision (TME) is the standard of cure [7]. However, some studies [8, 9] have reported better outcomes for the not negligible part of the patients who reach pathological complete response (pCR) after RTCT. In order to apply organ-preserving strategies, as well as to personalize treatments or to deescalate therapies [10], there is a great interest in stratifying the risk in patients with LARC, aimed at predicting pCR by exploiting various techniques which include radiomics [11]. Moreover, it would be of practical utility whether this could be accomplished by using the available CT images acquired for radiation therapy planning [12–14]. In this regard, we note that previous studies have assessed the potential role of CT imaging radiomics in rectal cancer both for contrast-enhanced [12, 13, 15–17] and noncontrast CT scans [14, 18].

Robustness of radiomic features relies on reproducibility and repeatability of their estimates considering different aspects of the radiomic workflow [19, 20]. Previous studies of CT imaging [21–24], as well as of MR [25–30] and NM [31–34] imaging, have assessed the reproducibility and repeatability of radiomic features estimation for various applications. Phantom and in vivo CT studies have reported dependence of radiomic feature estimates on various factors such as scanner type [35, 36], tube current [37–39], acquisition voxel size [21, 35], reconstruction kernel [40–43], and number of gray levels [21] or gray level discretization [35]. Shafiq-Ul-Hassan et al. [21], given that in clinical studies CT images are acquired using different voxel sizes, have suggested that resampling all image data sets with the same isotropic voxel size allows to reduce variability in radiomic features estimation. This specific preprocessing step can be accomplished by using different interpolation algorithms. However, the effect of the used interpolation algorithm on CT imaging radiomic feature estimate is usually not taken into account.

Only few studies have investigated the robustness of radiomic features from CT imaging in rectal cancer. For instance, Hu et al. [44] have studied feature stability in repeated CT acquisition, showing that features normalized to the tumor volume and those calculated as average over slices exhibit greater values of intraclass correlation coefficient (ICC) and concordance correlation coefficient (CCC) with respect to the unnormalized ones. Van Timmeren et al. [45] have compared two different test-retest situations, i.e., the analysis of repeated CT acquisitions after 15 minutes and few days in lung and rectal cancer, respectively. They have found that 446/542 features have a higher CCC for the test-retest analysis of the data set of patients with lung cancer than for patients with rectal cancer, showing the importance of controlling factors such as scanner, imaging protocol, reconstruction methods, and time points in a radiomic analysis.

Therefore, the aim of this study was to specifically assess, for the first time, the effect of preprocessing—in terms of resampling voxel size, interpolation algorithm, and bin width—on radiomic features estimation from CT imaging in patients with LARC.

## 2. Material and Methods

### 2.1. Patients and CT Imaging

Twenty representative patients with LARC were enrolled in this retrospective study approved by the internal review board. All patients underwent clinical CT imaging for preoperative radiotherapy. CT scanner (manufacturer/model) and acquisition parameters are reported in Table 1.

For each patient, the rectal gross tumor volume (GTV) was delineated (avoiding the inclusion of air regions) on CT images by a single experienced radiation oncologist, using the Eclipse treatment planning system (version 8.6, Varian, Palo Alto, CA). Then, a binary mask of the GTV region was created by employing 3D Slicer (version 4.10.2) [46].

### 2.2. Preprocessing of CT Images

A lower threshold of -500 HU was applied to the CT images, in order to account for partial volume effect and exclude voxels containing air from analyses.

The original isotropic voxel size (i.e., the cube root of the acquisition voxel volume) of CT imaging ranged approximately from 1.5 mm to 2 mm across patients. Therefore, in order to assess any effect of resampling voxel size on radiomic features estimation, CT images were resampled to isotropic voxels with size of 1 mm, 1.3 mm, 1.6 mm, 1.9 mm, 2.2 mm, and 2.5 mm. For each resampling voxel size, this was performed by using 10 different interpolation algorithms available in PyRadiomics (version 3.0) [47] (namely, BSpline (BS), BlackmanWindowedSinc (BL), CosineWindowedSinc (CWS), Gaussian (G), HammingWindowedSinc (HWS), LabelGaussian (LG), LanczosWindowedSinc (LWS), Linear (L), NearestNeighbor (NN), and WelchWindowedSinc (WWS)), at fixed bin width of 5 HU.

Furthermore, in order to assess any effect of bin width on radiomic features estimation, different bin width values of 3 HU, 4 HU, 5 HU, 6 HU, 7 HU, and 8 HU were employed. Indeed, for all patients, a bin width of 5 HU yielded a suggested number of quantization levels between 30 and 130 [4, 33, 48]. Accordingly, only bin width values close to 5 HU were considered. This was performed for different resampling voxel sizes (i.e., 1 mm, 1.3 mm, 1.6 mm, 1.9 mm, 2.2 mm, and 2.5 mm), at a fixed interpolation algorithm (i.e., BS).

All preprocessing of CT images was carried out by using the open source PyRadiomics library [47] (version 3.0.1) with Python (version 3.7.3).

### 2.3. Radiomic Features Estimation

Radiomic features estimation was performed through PyRadiomics (version 3.0.1) [47]. All but 9 radiomic features were calculated in compliance with the Image Biomarker Standardization Initiative (IBSI) [49]. For the remaining 9 radiomic features, 7 (namely, voxel volume, mesh volume, maximum probability, joint energy, sum squares, uniformity, and entropy) were calculated using IBSI formulas but presented different names, 1 (i.e., kurtosis) was in accordance with IBSI except for an offset value (i.e., 3), and 1 (i.e., total energy) was not defined by IBSI.

For each GTV region and preprocessing parameter combination (in terms of different resampling voxel sizes, interpolation algorithms, and bin widths), a total of 105 features, divided into 7 classes, were estimated. In particular, 14 shape features (shape class), 18 first-order features (first-order class), 22 gray level cooccurrence matrix features (GLCM class), 14 gray level dependence matrix features (GLDM class, with coarseness parameter *α* = 0), 16 gray level run length matrix features (GLRLM class), 16 gray level size zone features (GLSZM class), and 5 neighbouring gray tone difference matrix features (NGTDM class) were estimated. Second-order features estimation was performed according to the Chebyshev norm with a distance of 1 pixel. Only 3D versions of features were considered. GLCM and GLRLM features were computed from each 3D directional matrix (i.e., the 13 matrices identified by the 13 unique direction vectors within the 26 connected neighbouring voxels) and averaged over the 3D directions.

### 2.4. Statistical Analysis

In this study, four different effects on radiomic features estimation were assessed:

- For each resampling voxel size, with fixed bin width (i.e., 5 HU), effect of using different interpolation algorithms;
- For each resampling voxel size, with fixed interpolation algorithm (i.e., BS), effect of using different bin widths;
- For each bin width, with fixed interpolation algorithm (i.e., BS), effect of using different resampling voxel sizes;
- For each interpolation algorithm, with fixed bin width (i.e., 5 HU), effect of using different voxel sizes.

For each aforementioned effect of interest, any variability in radiomic feature estimate was assessed by means of ICC analysis [50, 51]. Specifically, the two-way mixed-effect model, with single rater and absolute agreement options, was applied to our data [50, 52]. Accordingly, the ICC coefficient was calculated as

(1) $$\begin{array}{c} \text{ICC}=\frac{{\text{MS}}_{\text{R}}-{\text{MS}}_{\text{E}}}{{\text{MS}}_{\text{R}}+\left(k-1\right){\text{MS}}_{\text{E}}+k/n\left({\text{MS}}_{\text{C}}-{\text{MS}}_{\text{E}}\right)}, \end{array}$$

where MS_R_ is the mean square for rows, MS_E_ is the mean square for error, MS_C_ is the mean square for columns, *n* is the number of subjects, and *k* is the number of raters, with ICC matrices realized considering each resampling voxel size, interpolation algorithm, or quantization bin width as a rater and each patient as a subject. ICC, which ranges between 0 (maximum variability) and 1 (minimum variability), expresses the variability of radiomic feature estimate associated with the effect of interest (i.e., resampling voxel size, interpolation algorithm, and bin width) with respect to the variance between subjects. Then, ICC values of radiomic features were nominally stratified as follows: poor (ICC ≤ 0.5), moderate (0.5 < ICC ≤ 0.75), good (0.75 < ICC ≤ 0.9), and excellent (0.9 < ICC ≤ 1) [36, 52, 53].

In order to better characterize the reproducibility of radiomic features estimation, an additional analysis of the coefficient of variation (CV) was performed. In particular, for each radiomic feature and patient, CV was calculated as the percentage ratio between standard deviation and mean values of feature estimates obtained by varying each considered preprocessing item (resampling voxel size, interpolation algorithm, or bin width) when the others were kept fixed.

Any linear correlation between radiomic features estimate and resampling voxel size or bin width was assessed through a repeated measures correlation analysis, namely, rmcorr [54]. This statistical technique accounts for nonindependence among observations (i.e., repeated measurements on the same subject with varying preprocessing) by using the analysis of covariance (ANCOVA) to adjust for individual differences.

Statistical analysis was performed by using R Studio (version 1.2.5033) and R (version 4.0.2) software packages [55].

## 3. Results

**Figure 1:**
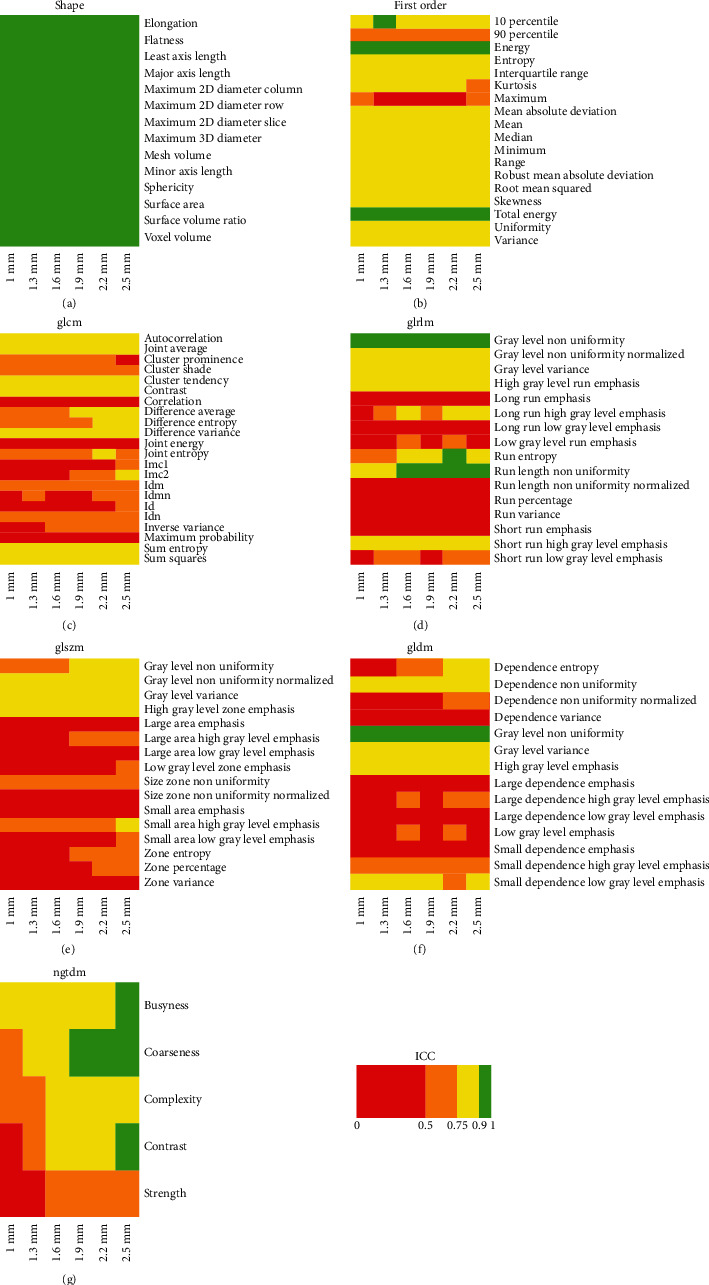
The heatmap of each radiomic feature class shows ICC results of effect A analysis, i.e., the assessment of variability in radiomic features estimate when using different interpolation algorithms, with fixed bin width (i.e., 5 HU) and for different isotropic resampling voxel sizes (i.e., 1, 1.3, 1.6, 1.9, 2.2, and 2.5 mm).

**Figure 2:**
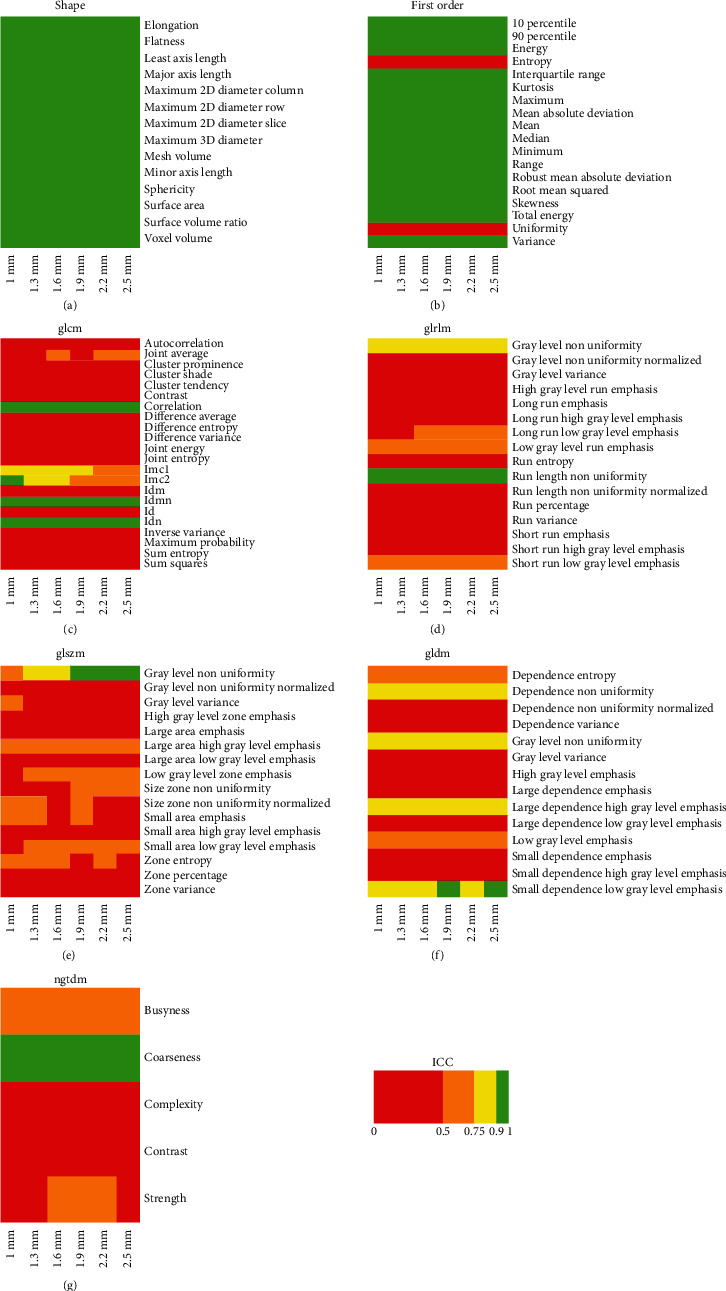
The heatmap of each radiomic feature class shows ICC results of effect B analysis, i.e., the assessment of variability in radiomic features estimate when using different bin widths, with fixed interpolation algorithm (i.e., BS) and for different isotropic resampling voxel sizes (i.e., 1, 1.3, 1.6, 1.9, 2.2, and 2.5 mm).

**Figure 3:**
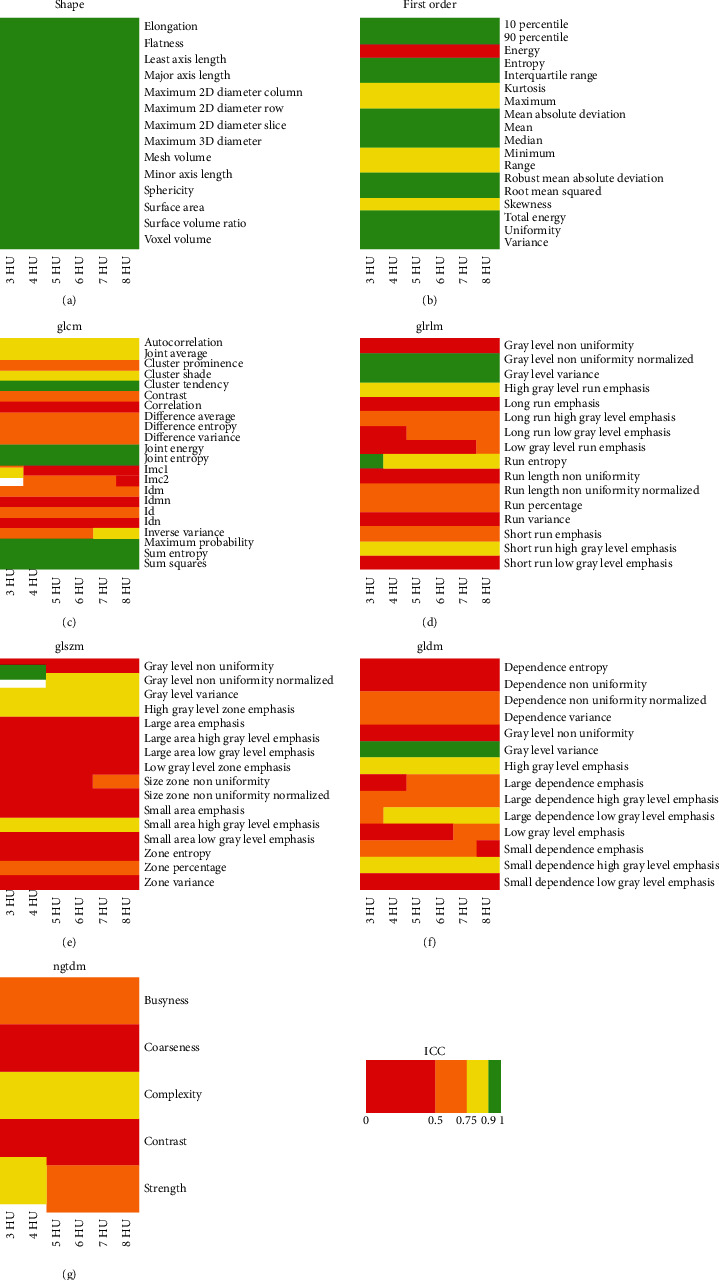
The heatmap of each radiomic feature class shows ICC results of effect C analysis, i.e., the assessment of variability in radiomic features estimate when using different resampling voxel sizes, with fixed interpolation algorithm (i.e., BS) and for different bin widths (i.e., 3, 4, 5, 6, 7, and 8 HU).

**Figure 4:**
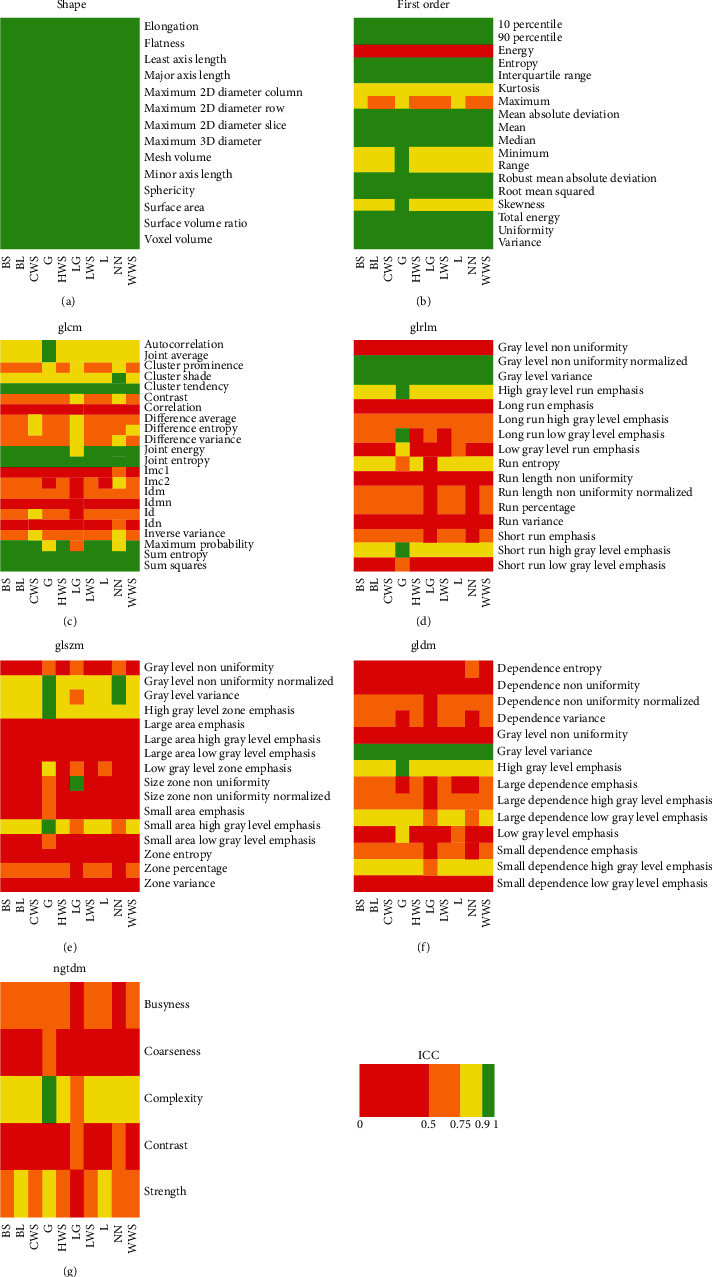
The heatmap of each radiomic feature class shows ICC results of effect D analysis, i.e., the assessment of variability in radiomic features estimate when using different resampling voxel sizes, with fixed bin width (i.e., 5 HU) and for different interpolation algorithms (i.e., BS, BL, CWS, G, HWS, LG, LWS, L, NN, and WWS).

ICC results for effects A, B, C, and D are reported in detail in Figures 1–4, respectively. ICC values were excellent for all features belonging to the shape class. In general, radiomic features belonging to the first-order class showed good or moderate ICC values, while features belonging to the other textural classes (GLCM, GLRLM, GLSZM, GLDM, and NGTDM) expressed moderate or poor ICC values.

**Figure 5:**
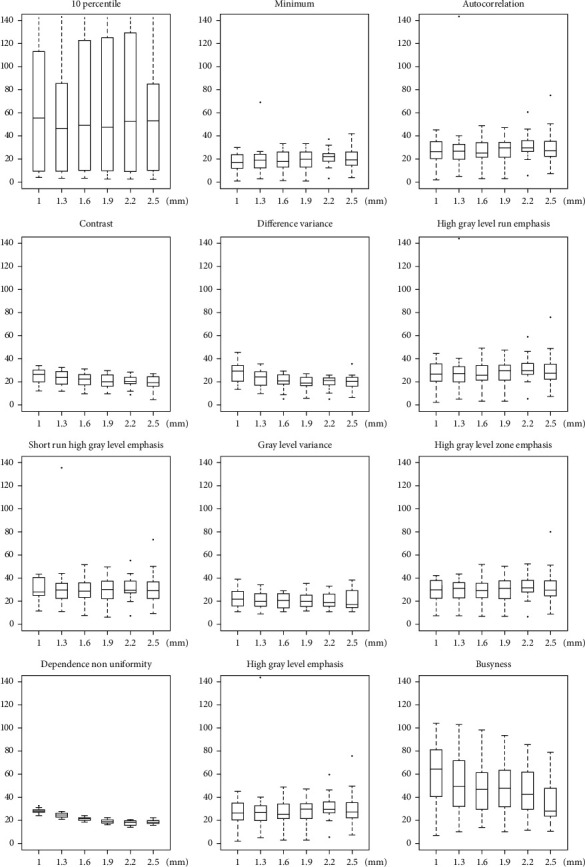
Box and whisker plots of CV (%) values for effect A of enrolled subjects. Radiomic features with both median (across subjects and different resampling voxel sizes) CV ≥ 15% and ICC ≥ 0.75 for each resampling voxel size (i.e., 1, 1.3, 1.6, 1.9, 2.2, and 2.5 mm) are shown.

**Figure 6:**
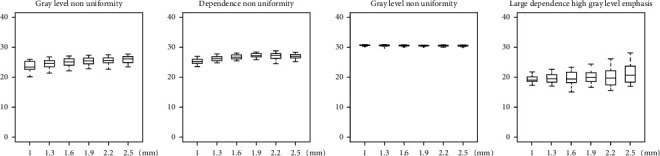
Box and whisker plots of CV (%) values for effect B of enrolled subjects. Radiomic features with both median (across subjects and different resampling voxel sizes) CV ≥ 15% and ICC ≥ 0.75 for each resampling voxel size (i.e., 1, 1.3, 1.6, 1.9, 2.2, and 2.5 mm) are shown.

**Figure 7:**
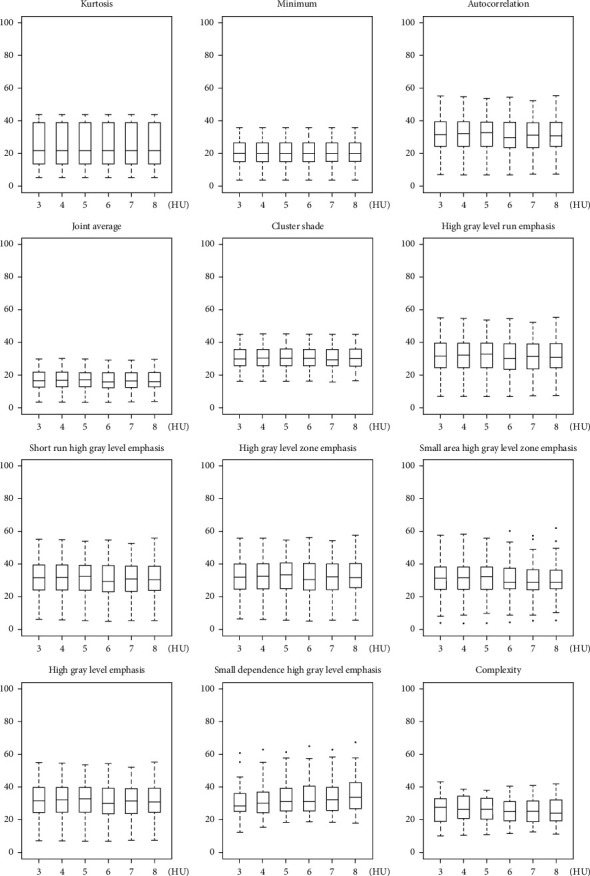
Box and whisker plots of CV (%) values for effect C of enrolled subjects. Radiomic features with both median (across subjects and different bin widths) CV ≥ 15% and ICC ≥ 0.75 for each bin width (i.e., 3, 4, 5, 6, 7, and 8 HU) are shown.

**Figure 8:**
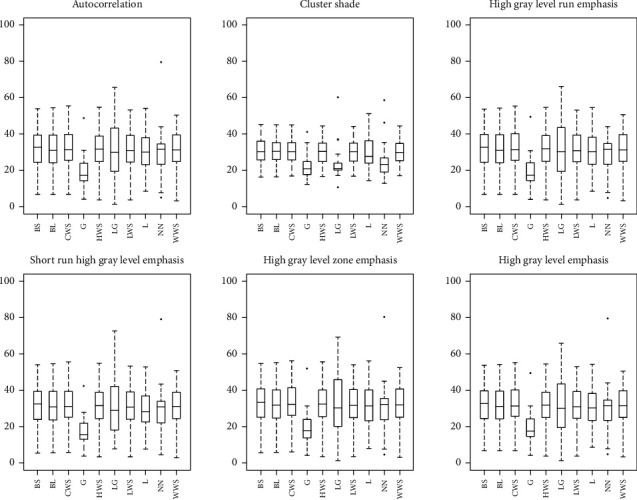
Box and whisker plots of CV (%) values for effect D of enrolled subjects. Radiomic features with both median (across subjects and different interpolation algorithms) CV ≥ 15% and ICC ≥ 0.75 for each interpolation algorithm (i.e., BS, BL, CWS, G, HWS, LG, LWS, L, NN, and WWS) are shown.

CV of radiomic features estimate for each effect of interest (i.e., A, B, C, and D) is summarized in Table 2, showing that for a number of features the variability associated with the four effects of interest can range up to 40% or more. Moreover, CV of radiomic features estimate for effect A, B, C, and D is reported in greater detail in Figures 5–8, respectively, showing features with both median (across subjects and resampling voxel sizes, bin widths, and interpolation algorithms, for effect A/B, C, and D, respectively) CV ≥ 15% and ICC ≥ 0.75.

The results of the analysis of the linear correlation between radiomic features estimate and bin width or resampling voxel size are reported in Tables 3 and 4, respectively. Most of the textural features (i.e., those belonging to GLCM, GLRLM, GLSZM, GLDM, or NGTDM classes) were characterized by a significant (*p* < 0.05, adjusted using Bonferroni correction) linear correlation with respect to both voxel size and bin width within the considered range of variation (i.e., 1-2.5 mm and 3-8 HU for voxel size and bin width, respectively).

## 4. Discussion

Recent studies have suggested a potential role of CT imaging radiomics in rectal cancer [11–18]. Bibault et al. [12] have presented a novel approach combining deep learning with clinical and pretreatment CT imaging radiomic features to build a model predicting complete pathologic response in a multicenter cohort of patients with locally advanced rectal cancer treated with neoadjuvant chemoradiation, followed by surgery. They have found that this model correctly predicted complete response after neoadjuvant rectal chemoradiotherapy in 80% of patients. In another study [14], pretreatment CT-based radiomic signatures were developed and validated in two independent cohorts. This imaging biomarker has proven to provide a promising way to predict complete pathologic response and select patients for nonoperative management. On the other hand, Hamerla et al. [13] have reported no evidence of added value of a radiomic model based on noncontrast CT scans for prediction of complete pathologic response in locally advanced rectal cancer. Vandendorpe et al. [15] have aimed to determine the value of baseline contrast-enhanced CT texture analysis in the prediction of downstaging in patients with locally advanced rectal cancer, calculating a radiomic score as a linear combination of radiomic features. By using a multivariable prognostic score that included this radiomic score and clinical factors, they have shown that this approach may lead to more personalized treatment for each patient. Wang et al. [18] have suggested that, by supervised modeling, radiomic features from radiotherapy CT imaging can potentially predict overall survival for locally advanced rectal cancer patients with neoadjuvant chemoradiation treatment. Moreover, Huang et al. [17] have developed and validated a radiomic signature from contrast-enhanced CT imaging as a complementary tool to differentiate high-grade from low-grade colorectal adenocarcinoma, with an area under the receiver operating characteristic curve of 0.725.

There is increasing evidence that preprocessing can someway impact the estimation of radiomic features derived from CT imaging [21–23], as well as from MR [25–28] and NM [31–33] imaging, in various clinical applications [19]. The degree and relevance of this effect can depend on imaging technique/modality and clinical application (e.g., anatomical region and lesion type). In this regard, Traverso et al. [19] have reviewed radiomic feature reproducibility and repeatability issues as reported by numerous research groups for different anatomical sites and various aspects of the radiomic workflow (i.e., image acquisition and reconstruction, image preprocessing, and feature extraction). They have submitted that further investigations are needed on these issues, possibly expanding the cohort of cancer types and providing details on feature extraction, image preprocessing, and statistical cutoff values used to distinguish stable features. To the best of our knowledge, no previous study has assessed the effect of preprocessing on CT-based radiomic features of locally advanced rectal cancer. Moreover, the effect of the interpolation algorithm was recently reported worthy of attention both in MR imaging radiomics of the LARC [25] and in PET imaging radiomics of oesophageal cancer [53]. Thus, we performed a rather comprehensive analysis, which considered multiple preprocessing elements such as resampling voxel size, interpolation algorithms, and bin widths.

We found that preprocessing can substantially bias the estimation of several CT imaging radiomic features in patients with LARC. In particular, the estimate of most textural features (i.e., features of GLCM, GLDM, GLRLM, GLSZM, and NGTDM classes) showed a relevant dependence (ICC ≤ 0.5) on interpolation algorithm (Figure 1), resampling voxel size (Figures 3 and 4), or bin width (Figure 2). Notably, except for a limited number of radiomic features, results of Figures 1–3 show that the degree of variability in radiomic features estimation due to interpolation algorithm/bin width and resampling voxel size is rather independent of resampling voxel size and bin width, respectively. However, in Figure 4, the heatmaps present a more pixelated appearance, indicating that the effect of resampling voxel size on textural features estimation can be more appreciably modulated by the used interpolation algorithm. Furthermore, for numerous textural features, a significant linear correlation between their estimates and bin width or resampling voxel size was observed (Tables 3 and 4).

As expected, based on ICC analysis, shape radiomic features did not show a relevant dependence on preprocessing (ICC ≥ 0.9), with median CVs for different resampling voxel sizes within 0.6%-3.1% (Table 2). Moreover, first-order radiomic features were characterized in general by higher ICC values than textural features when varying preprocessing. In this regard, we note that, except for entropy and uniformity features, PyRadiomics—according to IBSI recommendations [49]—calculates intensity-based first-order features using original images without discretization, as indicated by the null median CVs for different bin widths (see Table 2).

ICC analysis allows only to assess how relevant is the variability in radiomic features estimation due to preprocessing with respect to intersubject variability. Therefore, we also calculated the CV for each effect of interest (i.e., A, B, C, and D), in order to obtain additional information on absolute variation in radiomic features estimate due to different resampling voxel sizes, interpolation algorithms, and bin widths. We found that several textural features showed median CVs greater than 40% or more (Table 2). Furthermore, we revealed that even some radiomic features with high ICC values (≥0.75) can have a nonnegligible variability in terms of CV (≥15%) (Figures 5–8). In particular, for some effects of interest, CV values of single subjects can range up to 60% or more—this should be considered when comparing radiomic data from different studies. The results of Figure 8, regarding a small number of only 6 radiomic features, might suggest a potential reduction in median CV values across subjects when using the Gaussian interpolation algorithms with respect to the other interpolation algorithms. The explanation of this potential effect, which however does not necessarily hold true for all radiomic features (data not shown), appears not straightforward. Indeed, the use of different interpolation algorithms for resampling voxel size can modify radiomic characteristics of images in a manner rather complex and not easily predictable [56, 57].

In radiomic analyses, image interpolation at the same voxel size is a common and recommended practice (especially in retrospective studies) to reduce any heterogeneity in acquisition voxel size, while image discretization is required to make texture features estimation computationally less burdensome [31, 49]. Nonetheless, it should be noted that these preprocessing steps modify de facto acquired image data and likely their radiomic characteristics. In particular, the alteration of acquired image data due to preprocessing can actually yield a possible variation in the estimates of radiomic features when using different resampling voxel sizes and bin widths. Therefore, professionals and researchers executing or planning clinical or research studies should be aware of this important aspect of radiomics.

Given the revealed nonnegligible effect of preprocessing on the estimate of CT radiomic features in LARC, some caution regarding this aspect is recommended in clinical studies [23, 33, 37], mostly when considering textural radiomic features [58]. This emphasizes the importance of clearly identifying, assessing, and reporting all the processes involved in the applied radiomic workflow [3–5, 11, 19, 31].

## 5. Conclusions

In patients with LARC, the estimate of CT imaging-derived texture radiomic features, as well as of intensity-based first-order radiomic features to a lesser extent, is appreciably biased by resampling voxel size, interpolation algorithm, and bin width. Accordingly, toward optimization and standardization of radiomic methods, this should be taken into account when planning a clinical study, as well as when performing multicenter studies.

## Figures and Tables

**Table 1:** CT scanner and acquisition parameters.

Scanner manufacturer/model	GE/LightSpeed RT 16
Scan mode	Helical
Tube voltage (kVp)	120
Rotation time (s)	0.7
Tube load (mAs)	140-170
Slice thickness (mm)	5
Pixel size (mm)	0.82-1.27
Matrix	512×512

**Table 2:** CV of radiomic features estimate for each effect of interest (i.e., A, B, C, and D) reported as median (interquartile range) value. The median (interquartile range) CV was calculated across subjects and resampling voxel sizes, bin widths, and interpolation algorithms, for effect A/B, C, and D, respectively.

	A	B	C	D
Shape				
Elongation	0.0 (0.0)	0.0 (0.0)	1.2 (1.2)	1.2 (1.2)
Flatness	0.0 (0.0)	0.0 (0.0)	1.3 (0.7)	1.3 (0.7)
Least axis length	0.0 (0.0)	0.0 (0.0)	1.0 (0.8)	1.0 (0.8)
Major axis length	0.0 (0.0)	0.0 (0.0)	0.6 (0.7)	0.6 (0.7)
Maximum 2D diameter column	0.0 (0.0)	0.0 (0.0)	1.1 (0.5)	1.1 (0.5)
Maximum 2D diameter row	0.0 (0.0)	0.0 (0.0)	0.8 (0.8)	0.8 (0.8)
Maximum 2D diameter slice	0.0 (0.0)	0.0 (0.0)	0.9 (0.7)	0.9 (0.7)
Maximum 3D diameter	0.0 (0.0)	0.0 (0.0)	0.8 (0.4)	0.8 (0.4)
Mesh volume	0.0 (0.0)	0.0 (0.0)	1.7 (1.0)	1.7 (1.0)
Minor axis length	0.0 (0.0)	0.0 (0.0)	0.6 (0.6)	0.6 (0.6)
Sphericity	0.0 (0.0)	0.0 (0.0)	2.8 (0.7)	2.8 (0.7)
Surface area	0.0 (0.0)	0.0 (0.0)	3.1 (0.9)	3.1 (0.9)
Surface volume ratio	0.0 (0.0)	0.0 (0.0)	2.8 (0.7)	2.8 (0.7)
Voxel volume	0.0 (0.0)	0.0 (0.0)	1.6 (0.9)	1.6 (0.9)
First-order				
10 percentile	50.5 (103.0)	0.0 (0.0)	9.6 (27.1)	9.8 (27.7)
90 percentile	6.5 (4.0)	0.0 (0.0)	0.9 (0.4)	0.8 (1.0)
Energy	0.6 (0.5)	0.0 (0.0)	97.1 (0.4)	97.1 (0.4)
Entropy	3.1 (1.1)	11.8 (1.6)	0.6 (0.6)	0.6 (0.6)
Interquartile range	8.4 (5.5)	0.0 (0.0)	2.3 (1.1)	2.4 (1.5)
Kurtosis	23.2 (13.6)	0.0 (0.0)	21.8 (24.1)	18.8 (20.8)
Maximum	18.3 (7.0)	0.0 (0.0)	6.5 (1.7)	6.3 (3.8)
Mean absolute deviation	6.4 (4.9)	0.0 (0.0)	2.1 (1.7)	1.9 (1.5)
Mean	11.0 (15.8)	0.0 (0.0)	1.4 (1.8)	1.4 (1.8)
Median	6.8 (5.4)	0.0 (0.0)	1.0 (1.4)	1.2 (1.6)
Minimum	20.1 (11.3)	0.0 (0.0)	20.0 (10.8)	18.0 (12.3)
Range	16.3 (6.7)	0.0 (0.0)	14.8 (8.8)	14.3 (8.9)
Robust mean absolute deviation	7.7 (5.4)	0.0 (0.0)	1.7 (1.1)	1.7 (1.1)
Root mean squared	0.3 (0.3)	0.0 (0.0)	0.0 (0.0)	0.0 (0.0)
Skewness	15.5 (10.5)	0.0 (0.0)	12.3 (13.6)	10.3 (12.5)
Total energy	0.6 (0.5)	0.0 (0.0)	1.5 (0.9)	1.5 (1.0)
Uniformity	10.0 (3.2)	30.6 (0.3)	1.3 (1.1)	1.4 (1.4)
Variance	14.6 (9.5)	0.0 (0.0)	7.8 (5.9)	7.0 (6.2)
glcm				
Autocorrelation	27.9 (13.4)	69.8 (0.9)	31.2 (14.6)	30.4 (16.6)
Joint average	16.0 (8.8)	34.4 (0.5)	16.5 (8.9)	15.8 (9.3)
Cluster prominence	36.2 (33.7)	132.6 (0.2)	40.6 (10.9)	39.2 (18.2)
Cluster shade	26.6 (36.4)	104.4 (0.1)	30.4 (9.7)	27.2 (12.2)
Cluster tendency	12.6 (15.7)	70.8 (0.2)	13.2 (4.3)	11.9 (4.7)
Contrast	22.0 (8.9)	70.5 (0.5)	25.6 (8.4)	24.9 (11.2)
Correlation	20.7 (12.1)	0.4 (0.4)	29.8 (4.9)	28.1 (8.6)
Difference average	14.5 (5.9)	35.0 (0.1)	12.7 (3.8)	12.6 (5.0)
Difference entropy	6.5 (3.7)	14.0 (1.9)	5.6 (2.0)	5.5 (2.1)
Difference variance	22.1 (9.0)	70.1 (1.2)	27.1 (9.7)	26.1 (13.1)
Joint energy	39.8 (13.1)	55.0 (1.0)	7.7 (3.0)	8.3 (4.4)
Joint entropy	4.8 (2.0)	12.3 (2.3)	1.3 (0.7)	1.2 (0.8)
Imc1	36.1 (14.3)	7.3 (6.2)	24.7 (15.3)	23.8 (13.8)
Imc2	8.3 (4.7)	6.0 (5.9)	7.4 (5.2)	7.0 (4.4)
Idm	17.7 (3.7)	23.0 (2.5)	9.3 (3.5)	9.9 (4.0)
Idmn	0.1 (0.2)	0.0 (0.0)	0.2 (0.1)	0.2 (0.1)
Id	12.2 (3.0)	16.2 (1.5)	6.4 (2.3)	6.8 (3.0)
Idn	0.6 (0.6)	0.0 (0.0)	1.0 (0.3)	1.0 (0.4)
Inverse variance	11.9 (4.0)	20.1 (4.2)	6.8 (4.2)	6.8 (3.6)
Maximum probability	46.2 (16.4)	52.2 (3.7)	7.8 (3.5)	8.7 (5.4)
Sum entropy	2.4 (1.0)	10.1 (1.1)	1.6 (0.5)	1.5 (0.5)
Sum squares	14.1 (7.7)	70.7 (0.2)	7.2 (4.3)	6.7 (4.6)
glrlm				
Gray level nonuniformity	5.9 (3.2)	25.3 (2.2)	94.1 (1.9)	93.7 (3.3)
Gray level nonuniformity normalized	8.0 (3.1)	29.0 (0.8)	1.4 (1.3)	1.6 (1.5)
Gray level variance	13.0 (12.0)	68.3 (1.1)	8.1 (5.6)	7.4 (5.5)
High gray level run emphasis	28.3 (13.0)	70.0 (0.8)	31.4 (14.8)	30.4 (16.6)
Long run emphasis	26.0 (21.5)	13.6 (6.2)	10.1 (6.5)	10.5 (18.9)
Long run high gray level emphasis	30.5 (21.0)	56.0 (6.7)	36.6 (17.8)	41.8 (19.2)
Long run low gray level emphasis	73.0 (40.4)	52.6 (21.7)	43.2 (28.8)	40.5 (22.6)
Low gray level run emphasis	47.6 (32.6)	40.5 (18.1)	51.4 (26.2)	49.2 (20.3)
Run entropy	2.6 (1.8)	6.8 (0.9)	2.4 (1.2)	2.4 (1.3)
Run length non uniformity	11.4 (7.5)	11.2 (4.3)	90.6 (4.4)	90.1 (6.0)
Run length nonuniformity normalized	7.4 (5.2)	7.0 (2.5)	4.0 (2.2)	3.9 (2.5)
Run percentage	5.1 (3.5)	4.3 (1.8)	2.6 (1.4)	2.6 (2.1)
Run variance	65.8 (26.3)	40.9 (3.3)	35.2 (3.8)	35.2 (9.0)
Short run emphasis	4.0 (2.8)	3.0 (1.2)	1.8 (1.0)	1.8 (1.3)
Short run high gray level emphasis	29.3 (14.2)	72.8 (1.4)	31.1 (14.8)	29.8 (17.2)
Short run low gray level emphasis	44.4 (31.5)	38.4 (17.0)	52.6 (24.8)	51.4 (20.5)
glszm				
Gray level nonuniformity	25.6 (10.2)	13.6 (13.7)	58.5 (17.6)	56.6 (16.8)
Gray level nonuniformity normalized	8.6 (3.5)	23.2 (3.3)	6.7 (3.5)	6.3 (3.7)
Gray level variance	19.9 (11.6)	49.4 (6.6)	16.0 (8.9)	13.9 (8.4)
High gray level zone emphasis	30.4 (13.5)	73.0 (1.2)	31.8 (15.4)	31.0 (17.6)
Large area emphasis	72.1 (37.7)	89.9 (12.1)	139.4 (15.1)	138.8 (14.6)
Large area high gray level emphasis	64.1 (51.2)	55.9 (15.9)	146.5 (17.6)	146.4 (12.3)
Large area low gray level emphasis	105.2 (56.7)	115.5 (9.0)	124.0 (25.6)	126.2 (29.4)
Low gray level zone emphasis	49.4 (25.0)	47.5 (13.9)	48.7 (17.8)	46.0 (16.7)
Size zone nonuniformity	39.7 (7.1)	45.5 (14.5)	52.4 (18.7)	52.0 (18.4)
Size zone nonuniformity normalized	22.6 (4.2)	8.5 (5.5)	12.5 (5.0)	13.7 (7.9)
Small area emphasis	32.9 (8.7)	4.4 (2.9)	6.7 (3.0)	7.4 (5.0)
Small area high gray level emphasis	45.3 (12.0)	78.4 (3.3)	29.9 (13.4)	30.3 (17.3)
Small area low gray level emphasis	50.8 (29.5)	45.8 (12.9)	53.5 (16.0)	54.4 (22.7)
Zone entropy	4.5 (1.8)	3.6 (1.7)	6.3 (1.8)	6.0 (1.5)
Zone percentage	29.4 (10.1)	38.0 (13.3)	25.9 (9.8)	26.4 (13.0)
Zone variance	74.8 (38.9)	90.3 (12.4)	140.6 (14.4)	140.2 (13.8)
gldm				
Dependence entropy	2.7 (1.4)	2.5 (0.3)	3.6 (0.7)	3.7 (1.1)
Dependence nonuniformity	20.6 (5.7)	26.8 (1.5)	84.6 (5.4)	83.8 (6.9)
Dependence nonuniformity normalized	20.6 (5.7)	26.8 (1.5)	12.7 (4.3)	13.6 (5.5)
Dependence variance	62.8 (12.9)	40.9 (2.4)	21.7 (8.5)	24.2 (9.2)
Gray level nonuniformity	10.0 (3.2)	30.6 (0.3)	97.1 (0.7)	97.0 (0.7)
Gray level variance	14.5 (9.5)	70.8 (0.2)	7.8 (5.9)	7.1 (6.2)
High gray level emphasis	28.0 (13.3)	69.8 (0.9)	31.4 (14.8)	30.4 (16.5)
Large dependence emphasis	57.1 (19.1)	42.4 (1.6)	26.4 (6.5)	27.9 (10.1)
Large dependence high gray level emphasis	55.1 (34.4)	19.6 (2.9)	47.5 (12.9)	50.2 (16.2)
Large dependence low gray level emphasis	98.0 (42.6)	84.3 (3.4)	25.2 (17.5)	25.8 (17.6)
Low gray level emphasis	45.8 (31.2)	40.2 (18.9)	51.0 (26.7)	49.1 (21.4)
Small dependence emphasis	31.8 (6.1)	33.3 (9.7)	22.9 (7.0)	23.1 (6.6)
Small dependence high gray level emphasis	46.1 (12.6)	102.4 (7.9)	31.4 (14.4)	31.4 (15.1)
Small dependence low gray level emphasis	23.6 (13.5)	14.3 (9.7)	67.9 (16.4)	67.6 (16.1)
ngtdm				
Busyness	46.2 (35.3)	50.6 (4.1)	51.7 (11.2)	51.8 (14.9)
Coarseness	22.8 (16.7)	0.5 (0.3)	68.1 (5.8)	68.5 (6.4)
Complexity	34.2 (8.6)	89.8 (5.8)	25.9 (13.3)	22.4 (14.2)
Contrast	23.1 (13.8)	48.3 (7.5)	49.7 (10.7)	48.5 (15.9)
Strength	37.2 (19.7)	51.8 (7.5)	31.8 (11.5)	32.0 (17.8)

**Table 3:** *R* coefficient of linear correlation between radiomic features estimates and bin width for different resampling voxel sizes (i.e., 1, 1.3, 1.6, 1.9, 2.2, and 2.5 mm). Significant (p < 0.05, adjusted for Bonferroni correction) correlations are highlighted in bold.

	1	1.3	1.6	1.9	2.2	2.5
Shape						
Elongation	0.10	-0.11	0.02	0.09	0.05	-0.12
Flatness	0.16	0.15	0.16	0.10	0.11	0.19
Least axis length	-0.05	0.15	0.04	0.19	0.15	0.16
Major axis length	-0.03	0.19	0.04	0.02	-0.06	0.19
Maximum 2D diameter column	0.13	0.11	0.03	-0.11	-0.07	-0.07
Maximum 2D diameter row	0.15	0.21	-0.09	0.20	0.12	0.01
Maximum 2D diameter slice	0.22	0.00	0.08	0.20	0.19	0.20
Maximum 3D diameter	0.19	0.06	0.09	0.22	-0.02	-0.01
Mesh volume	0.06	0.12	0.02	0.15	0.10	0.07
Minor axis length	-0.03	-0.10	0.17	0.10	0.18	0.13
Sphericity	-0.17	-0.20	-0.07	0.06	-0.08	-0.10
Surface area	0.01	0.04	0.17	0.10	0.14	0.05
Surface volume ratio	-0.03	0.01	0.10	0.12	0.12	-0.18
Voxel volume	0.06	0.01	0.11	0.04	0.04	0.07
First-order						
10 percentile	0.01	0.06	0.05	0.05	-0.06	-0.05
90 percentile	-0.21	-0.21	-0.23	-0.20	-0.20	-0.20
Energy	0.10	0.17	0.10	0.18	0.13	0.14
Entropy	**-0.99**	**-0.99**	**-0.99**	**-0.99**	**-0.99**	**-0.99**
Interquartile range	0.13	-0.20	0.07	0.08	0.01	-0.10
Kurtosis	0.20	0.02	0.08	0.18	0.12	0.20
Maximum	-0.11	0.13	0.12	-0.14	-0.07	-0.13
Mean absolute deviation	0.00	0.03	0.05	0.12	-0.16	-0.04
Mean	-0.21	-0.18	-0.05	0.09	-0.16	-0.13
Median	0.10	0.09	0.09	0.02	0.13	0.11
Minimum	-0.09	-0.21	-0.16	-0.09	-0.08	-0.20
Range	-0.04	0.19	-0.07	0.15	0.14	0.08
Robust mean absolute deviation	-0.05	0.02	0.08	0.09	-0.18	-0.18
Root mean squared	0.09	0.14	-0.16	-0.15	0.15	0.15
Skewness	-0.17	-0.11	0.03	-0.07	-0.10	-0.22
Total energy	0.10	-0.02	0.07	0.12	0.12	0.15
Uniformity	**0.98**	**0.98**	**0.98**	**0.98**	**0.97**	**0.97**
Variance	0.00	-0.03	0.07	-0.09	-0.02	0.09
glcm						
Autocorrelation	**-0.77**	**-0.76**	**-0.75**	**-0.76**	**-0.71**	**-0.72**
Joint average	**-0.90**	**-0.90**	**-0.89**	**-0.90**	**-0.86**	**-0.89**
Cluster prominence	**-0.55**	**-0.55**	**-0.54**	**-0.56**	**-0.52**	**-0.55**
Cluster shade	**0.62**	**0.62**	**0.62**	**0.63**	**0.59**	**0.62**
Cluster tendency	**-0.76**	**-0.76**	**-0.78**	**-0.77**	**-0.75**	**-0.77**
Contrast	**-0.81**	**-0.81**	**-0.81**	**-0.80**	**-0.77**	**-0.79**
Correlation	**-0.86**	**-0.82**	**-0.78**	**-0.73**	**-0.64**	**-0.54**
Difference average	**-0.94**	**-0.94**	**-0.93**	**-0.93**	**-0.93**	**-0.93**
Difference entropy	**-0.99**	**-0.99**	**-0.99**	**-0.99**	**-0.99**	**-0.99**
Difference variance	**-0.78**	**-0.78**	**-0.78**	**-0.77**	**-0.74**	**-0.77**
Joint energy	**0.94**	**0.93**	**0.92**	**0.92**	**0.91**	**0.92**
Joint entropy	**-0.99**	**-0.99**	**-0.99**	**-0.99**	**-0.99**	**-0.99**
Imc1	**-0.93**	**-0.66**	0.14	**0.57**	**0.70**	**0.76**
Imc2	**-0.84**	**-0.85**	**-0.88**	**-0.92**	**-0.94**	**-0.96**
Idm	**0.99**	**0.99**	**0.99**	**0.99**	**0.99**	**0.99**
Idmn	-0.13	0.20	0.23	0.19	0.37	0.25
Id	**0.99**	**0.99**	**0.99**	**0.99**	**0.99**	**0.99**
Idn	**0.64**	**0.63**	**0.63**	**0.55**	**0.61**	**0.53**
Inverse variance	**0.98**	**0.99**	**0.99**	**0.99**	**0.99**	**0.99**
Maximum probability	**0.95**	**0.94**	**0.92**	**0.93**	**0.93**	**0.92**
Sum entropy	**-0.99**	**-0.99**	**-0.99**	**-0.99**	**-0.99**	**-0.99**
Sum squares	**-0.77**	**-0.78**	**-0.79**	**-0.78**	**-0.76**	**-0.78**
glrlm						
Gray level nonuniformity	**0.75**	**0.75**	**0.75**	**0.75**	**0.75**	**0.75**
Gray level nonuniformity normalized	**0.98**	**0.98**	**0.98**	**0.98**	**0.98**	**0.98**
Gray level variance	**-0.77**	**-0.77**	**-0.78**	**-0.77**	**-0.76**	**-0.78**
High gray level run emphasis	**-0.77**	**-0.76**	**-0.75**	**-0.76**	**-0.71**	**-0.72**
Long run emphasis	**0.95**	**0.95**	**0.94**	**0.95**	**0.95**	**0.95**
Long run high gray level emphasis	**-0.78**	**-0.77**	**-0.75**	**-0.77**	**-0.71**	**-0.72**
Long run low gray level emphasis	**0.69**	**0.72**	**0.62**	**0.68**	**0.67**	**0.73**
Low gray level run emphasis	**0.73**	**0.74**	**0.66**	**0.70**	**0.67**	**0.74**
Run entropy	**-0.97**	**-0.98**	**-0.98**	**-0.98**	**-0.98**	**-0.98**
Run length nonuniformity	**-0.76**	**-0.75**	**-0.75**	**-0.75**	**-0.74**	**-0.74**
Run length nonuniformity normalized	**-0.99**	**-0.99**	**-0.99**	**-0.99**	**-0.98**	**-0.98**
Run percentage	**-0.99**	**-0.98**	**-0.98**	**-0.98**	**-0.98**	**-0.97**
Run variance	**0.94**	**0.94**	**0.94**	**0.94**	**0.94**	**0.94**
Short run emphasis	**-0.98**	**-0.98**	**-0.98**	**-0.98**	**-0.98**	**-0.97**
Short run high gray level emphasis	**-0.77**	**-0.76**	**-0.75**	**-0.76**	**-0.70**	**-0.72**
Short run low gray level emphasis	**0.74**	**0.74**	**0.67**	**0.70**	**0.66**	**0.74**
glszm						
Gray level nonuniformity	**-0.72**	**-0.70**	**-0.66**	**-0.63**	**-0.58**	**-0.51**
Gray level nonuniformity normalized	**0.95**	**0.96**	**0.96**	**0.97**	**0.96**	**0.96**
Gray level variance	**-0.79**	**-0.80**	**-0.80**	**-0.80**	**-0.78**	**-0.81**
High gray level zone emphasis	**-0.77**	**-0.76**	**-0.75**	**-0.76**	**-0.70**	**-0.71**
Large area emphasis	**0.53**	**0.55**	**0.56**	**0.56**	**0.55**	**0.56**
Large area high gray level emphasis	**0.63**	**0.62**	**0.65**	**0.57**	**0.55**	**0.53**
Large area low gray level emphasis	**0.44**	**0.45**	**0.41**	**0.44**	**0.50**	**0.45**
Low gray level zone emphasis	**0.80**	**0.74**	**0.72**	**0.75**	**0.72**	**0.76**
Size zone nonuniformity	**-0.80**	**-0.80**	**-0.80**	**-0.80**	**-0.81**	**-0.80**
Size zone nonuniformity normalized	**-0.94**	**-0.96**	**-0.92**	**-0.90**	**-0.83**	**-0.80**
Small area emphasis	**-0.91**	**-0.95**	**-0.92**	**-0.90**	**-0.84**	**-0.81**
Small area high gray level emphasis	**-0.76**	**-0.75**	**-0.74**	**-0.75**	**-0.69**	**-0.71**
Small area low gray level emphasis	**0.84**	**0.70**	**0.70**	**0.77**	**0.72**	**0.75**
Zone entropy	**-0.95**	**-0.98**	**-0.97**	**-0.97**	**-0.97**	**-0.97**
Zone percentage	**-0.93**	**-0.95**	**-0.96**	**-0.97**	**-0.97**	**-0.97**
Zone variance	**0.53**	**0.55**	**0.56**	**0.56**	**0.54**	**0.56**
gldm						
Dependence entropy	**-0.99**	**-0.99**	**-0.99**	**-0.99**	**-0.98**	**-0.98**
Dependence nonuniformity	**-0.79**	**-0.79**	**-0.79**	**-0.80**	**-0.80**	**-0.80**
Dependence nonuniformity normalized	**-0.95**	**-0.95**	**-0.95**	**-0.95**	**-0.96**	**-0.96**
Dependence variance	**0.97**	**0.96**	**0.95**	**0.95**	**0.95**	**0.95**
Gray level nonuniformity	**0.74**	**0.74**	**0.74**	**0.74**	**0.74**	**0.74**
Gray level variance	**-0.76**	**-0.77**	**-0.78**	**-0.77**	**-0.75**	**-0.78**
High gray level emphasis	**-0.77**	**-0.76**	**-0.75**	**-0.76**	**-0.71**	**-0.72**
Large dependence emphasis	**0.96**	**0.95**	**0.94**	**0.94**	**0.94**	**0.94**
Large dependence high gray level emphasis	**-0.82**	**-0.79**	**-0.77**	**-0.80**	**-0.73**	**-0.71**
Large dependence low gray level emphasis	**0.68**	**0.70**	**0.58**	**0.65**	**0.64**	**0.67**
Low gray level emphasis	**0.73**	**0.74**	**0.65**	**0.69**	**0.66**	**0.73**
Small dependence emphasis	**-0.95**	**-0.96**	**-0.97**	**-0.97**	**-0.97**	**-0.97**
Small dependence high gray level emphasis	**-0.70**	**-0.71**	**-0.70**	**-0.71**	**-0.67**	**-0.69**
Small dependence low gray level emphasis	**0.81**	**0.62**	**0.63**	**0.71**	**0.48**	**0.68**
ngtdm						
Busyness	**0.56**	**0.55**	**0.55**	**0.53**	**0.61**	**0.61**
Coarseness	**-0.74**	-0.38	0.14	0.28	0.25	0.38
Complexity	**-0.70**	**-0.69**	**-0.68**	**-0.68**	**-0.67**	**-0.69**
Contrast	**-0.84**	**-0.83**	**-0.83**	**-0.75**	**-0.80**	**-0.81**
Strength	**-0.78**	**-0.79**	**-0.77**	**-0.78**	**-0.78**	**-0.82**

**Table 4:** *R* coefficient of linear correlation between radiomic feature estimates and resampling voxel sizes for different bin widths (i.e., 3, 4, 5, 6, 7, and 8 HU). Significant (*p* < 0.05, adjusted for Bonferroni correction) correlations are highlighted in bold.

	3	4	5	6	7	8
Shape						
Elongation	-0.04	-0.04	-0.04	-0.04	-0.04	-0.04
Flatness	-0.09	-0.09	-0.09	-0.09	-0.09	-0.09
Least axis length	-0.04	-0.04	-0.04	-0.04	-0.04	-0.04
Major axis length	0.03	0.03	0.03	0.03	0.03	0.03
Maximum 2D diameter column	-0.23	-0.23	-0.23	-0.23	-0.23	-0.23
Maximum 2D diameter row	-0.21	-0.21	-0.21	-0.21	-0.21	-0.21
Maximum 2D diameter slice	-0.32	-0.32	-0.32	-0.32	-0.32	-0.32
Maximum 3D diameter	-0.27	-0.27	-0.27	-0.27	-0.27	-0.27
Mesh volume	-0.06	-0.06	-0.06	-0.06	-0.06	-0.06
Minor axis length	0.06	0.06	0.06	0.06	0.06	0.06
Sphericity	**0.95**	**0.95**	**0.95**	**0.95**	**0.95**	**0.95**
Surface area	**-0.85**	**-0.85**	**-0.85**	**-0.85**	**-0.85**	**-0.85**
Surface volume ratio	**-0.86**	**-0.86**	**-0.86**	**-0.86**	**-0.86**	**-0.86**
Voxel volume	0.12	0.12	0.12	0.12	0.12	0.12
First-order						
10 percentile	0.06	0.06	0.06	0.06	0.06	0.06
90 percentile	0.09	0.09	0.09	0.09	0.09	0.09
Energy	**-0.69**	**-0.69**	**-0.69**	**-0.69**	**-0.69**	**-0.69**
Entropy	**-0.41**	-0.34	-0.30	-0.25	-0.23	-0.20
Interquartile range	0.04	0.04	0.04	0.04	0.04	0.04
Kurtosis	-0.15	-0.15	-0.15	-0.15	-0.15	-0.15
Maximum	**-0.43**	**-0.43**	**-0.43**	**-0.43**	**-0.43**	**-0.43**
Mean absolute deviation	-0.07	-0.07	-0.07	-0.07	-0.07	-0.07
Mean	0.12	0.12	0.12	0.12	0.12	0.12
Median	-0.02	-0.02	-0.02	-0.02	-0.02	-0.02
Minimum	**0.57**	**0.57**	**0.57**	**0.57**	**0.57**	**0.57**
Range	**-0.60**	**-0.60**	**-0.60**	**-0.60**	**-0.60**	**-0.60**
Robust mean absolute deviation	0.05	0.05	0.05	0.05	0.05	0.05
Root mean squared	0.12	0.12	0.12	0.12	0.12	0.12
Skewness	0.17	0.17	0.17	0.17	0.17	0.17
Total energy	0.13	0.13	0.13	0.13	0.13	0.13
Uniformity	0.15	0.08	0.05	0.06	0.07	0.04
Variance	-0.12	-0.12	-0.12	-0.12	-0.12	-0.12
glcm						
Autocorrelation	**-0.53**	**-0.53**	**-0.53**	**-0.53**	**-0.53**	**-0.53**
Joint average	**-0.56**	**-0.56**	**-0.57**	**-0.56**	**-0.56**	**-0.56**
Cluster prominence	**-0.52**	**-0.52**	**-0.52**	**-0.52**	**-0.52**	**-0.52**
Cluster shade	**0.56**	**0.56**	**0.56**	**0.56**	**0.55**	**0.56**
Cluster tendency	**-0.69**	**-0.69**	**-0.69**	**-0.69**	**-0.69**	**-0.69**
Contrast	**0.77**	**0.77**	**0.77**	**0.77**	**0.76**	**0.76**
Correlation	**-0.98**	**-0.98**	**-0.98**	**-0.98**	**-0.98**	**-0.98**
Difference average	**0.89**	**0.89**	**0.89**	**0.89**	**0.88**	**0.88**
Difference entropy	**0.92**	**0.92**	**0.92**	**0.92**	**0.92**	**0.92**
Difference variance	**0.73**	**0.73**	**0.73**	**0.73**	**0.73**	**0.73**
Joint energy	**-0.45**	**-0.68**	**-0.72**	**-0.76**	**-0.73**	**-0.77**
Joint entropy	-0.36	-0.02	0.24	**0.42**	**0.51**	**0.58**
Imc1	0.01	**0.43**	**0.64**	**0.74**	**0.80**	**0.83**
Imc2	0.01	-0.35	**-0.56**	**-0.68**	**-0.76**	**-0.81**
Idm	**-0.93**	**-0.93**	**-0.93**	**-0.93**	**-0.92**	**-0.92**
Idmn	**-0.76**	**-0.76**	**-0.76**	**-0.75**	**-0.76**	**-0.77**
Id	**-0.93**	**-0.93**	**-0.93**	**-0.93**	**-0.93**	**-0.93**
Idn	**-0.81**	**-0.82**	**-0.82**	**-0.81**	**-0.82**	**-0.82**
Inverse variance	**-0.92**	**-0.92**	**-0.91**	**-0.91**	**-0.89**	**-0.87**
Maximum probability	0.17	-0.29	**-0.44**	**-0.42**	**-0.46**	**-0.64**
Sum entropy	**-0.92**	**-0.91**	**-0.90**	**-0.90**	**-0.89**	**-0.89**
Sum squares	-0.21	-0.21	-0.21	-0.21	-0.21	-0.21
glrlm						
Gray level nonuniformity	**-0.66**	**-0.66**	**-0.66**	**-0.66**	**-0.67**	**-0.67**
Gray level nonuniformity normalized	0.24	0.22	0.23	0.27	0.30	0.32
Gray level variance	-0.17	-0.19	-0.22	-0.23	-0.25	-0.27
High gray level run emphasis	**-0.53**	**-0.53**	**-0.53**	**-0.53**	**-0.53**	**-0.52**
Long run emphasis	**-0.92**	**-0.91**	**-0.91**	**-0.91**	**-0.90**	**-0.90**
Long run high gray level emphasis	**-0.63**	**-0.66**	**-0.68**	**-0.70**	**-0.71**	**-0.73**
Long run low gray level emphasis	**0.73**	**0.68**	**0.62**	**0.54**	**0.49**	**0.43**
Low gray level run emphasis	**0.74**	**0.72**	**0.68**	**0.63**	**0.62**	**0.59**
Run entropy	**-0.93**	**-0.94**	**-0.95**	**-0.95**	**-0.95**	**-0.95**
Run length nonuniformity	**-0.71**	**-0.71**	**-0.72**	**-0.72**	**-0.73**	**-0.73**
Run length nonuniformity normalized	**0.94**	**0.94**	**0.95**	**0.95**	**0.95**	**0.95**
Run percentage	**0.94**	**0.94**	**0.95**	**0.95**	**0.95**	**0.95**
Run variance	**-0.91**	**-0.90**	**-0.90**	**-0.90**	**-0.89**	**-0.89**
Short run emphasis	**0.94**	**0.94**	**0.94**	**0.94**	**0.94**	**0.94**
Short run high gray level emphasis	**-0.50**	**-0.50**	**-0.49**	**-0.48**	**-0.47**	**-0.46**
Short run low gray level emphasis	**0.75**	**0.72**	**0.69**	**0.64**	**0.64**	**0.60**
glszm						
Gray level nonuniformity	**-0.75**	**-0.78**	**-0.81**	**-0.83**	**-0.84**	**-0.85**
Gray level nonuniformity normalized	**0.78**	**0.85**	**0.86**	**0.86**	**0.86**	**0.87**
Gray level variance	**-0.55**	**-0.59**	**-0.63**	**-0.64**	**-0.66**	**-0.65**
High gray level zone emphasis	**-0.51**	**-0.50**	**-0.49**	**-0.48**	**-0.47**	**-0.46**
Large area emphasis	**-0.42**	**-0.44**	**-0.46**	**-0.44**	**-0.44**	**-0.44**
Large area high gray level emphasis	**-0.50**	**-0.50**	**-0.51**	**-0.51**	**-0.51**	**-0.51**
Large area low gray level emphasis	-0.38	-0.39	**-0.40**	-0.39	-0.39	-0.39
Low gray level zone emphasis	**0.76**	**0.73**	**0.71**	**0.66**	**0.65**	**0.61**
Size zone nonuniformity	**-0.81**	**-0.82**	**-0.81**	**-0.79**	**-0.77**	**-0.74**
Size zone nonuniformity normalized	**0.95**	**0.93**	**0.92**	**0.93**	**0.93**	**0.93**
Small area emphasis	**0.94**	**0.91**	**0.90**	**0.90**	**0.90**	**0.90**
Small area high gray level emphasis	-0.36	-0.34	-0.31	-0.27	-0.24	-0.22
Small area low gray level emphasis	**0.75**	**0.73**	**0.73**	**0.67**	**0.68**	**0.64**
Zone entropy	**-0.97**	**-0.97**	**-0.97**	**-0.97**	**-0.97**	**-0.97**
Zone percentage	**0.96**	**0.96**	**0.96**	**0.96**	**0.95**	**0.95**
Zone variance	**-0.42**	**-0.44**	**-0.46**	**-0.44**	**-0.44**	**-0.44**
gldm						
Dependence entropy	**-0.97**	**-0.97**	**-0.98**	**-0.97**	**-0.98**	**-0.98**
Dependence nonuniformity	**-0.73**	**-0.72**	**-0.72**	**-0.72**	**-0.72**	**-0.72**
Dependence nonuniformity normalized	**0.94**	**0.94**	**0.93**	**0.92**	**0.92**	**0.91**
Dependence variance	**-0.92**	**-0.92**	**-0.93**	**-0.93**	**-0.92**	**-0.93**
Gray level nonuniformity	**-0.65**	**-0.65**	**-0.65**	**-0.65**	**-0.65**	**-0.65**
Gray level variance	-0.12	-0.12	-0.12	-0.12	-0.12	-0.12
High gray level emphasis	**-0.53**	**-0.53**	**-0.53**	**-0.53**	**-0.53**	**-0.53**
Large dependence emphasis	**-0.91**	**-0.91**	**-0.91**	**-0.92**	**-0.91**	**-0.92**
Large dependence high gray level emphasis	**-0.76**	**-0.76**	**-0.76**	**-0.76**	**-0.75**	**-0.76**
Large dependence low gray level emphasis	0.31	0.18	0.14	0.11	0.09	0.09
Low gray level emphasis	**0.74**	**0.71**	**0.68**	**0.62**	**0.62**	**0.58**
Small dependence emphasis	**0.97**	**0.96**	**0.96**	**0.96**	**0.96**	**0.95**
Small dependence high gray level emphasis	-0.03	0.06	0.13	0.19	0.24	0.26
Small dependence low gray level emphasis	**0.75**	**0.74**	**0.74**	**0.67**	**0.71**	**0.68**
ngtdm						
Busyness	**-0.49**	**-0.49**	**-0.50**	**-0.50**	**-0.50**	**-0.50**
Coarseness	**0.77**	**0.77**	**0.77**	**0.76**	**0.76**	**0.76**
Complexity	**-0.67**	**-0.65**	**-0.62**	**-0.60**	**-0.56**	**-0.55**
Contrast	**0.82**	**0.82**	**0.84**	**0.84**	**0.84**	**0.85**
Strength	**0.54**	**0.54**	**0.53**	**0.56**	**0.55**	**0.57**

## Data Availability

The data used to support the findings of this study are available from the corresponding author upon request.
